# Ovarian Cancer Susceptibility and Chemosensitivity to KRAS Modulation

**DOI:** 10.3390/ijms27031571

**Published:** 2026-02-05

**Authors:** Alexandra Maria Psaras, Steven J. McKay, Janelle Vasquez Vilela, Eddison Ospina Sanchez, Marina G. Cintrón, Kayla K. Elder, Tracy A. Brooks

**Affiliations:** Department of Pharmaceutical Sciences, School of Pharmacy and Pharmaceutical Sciences, Binghamton University, Binghamton, NY 13902, USA; psarra.alexandra@gmail.com (A.M.P.); smckay3@binghamton.edu (S.J.M.); jv800@gsbs.rutgers.edu (J.V.V.); eospina1@binghamton.edu (E.O.S.); mcintro3@binghamton.edu (M.G.C.); kelder1@binghamton.edu (K.K.E.)

**Keywords:** KRAS, ovarian cancer, chemosensitization, paclitaxel, cisplatin, 3D spheroids, drug resistance, combination therapy

## Abstract

KRAS is frequently amplified or overexpressed in ovarian cancer and represents a potential therapeutic target for overcoming chemoresistance. We employed complementary approaches—CRISPR/Cas9 gene editing, Tet-ON inducible knockdown, polypurine reverse Hoogsteen hairpin (PPRH) oligonucleotides, and the pan-KRAS inhibitor BI2865—to investigate whether KRAS modulation enhances chemotherapeutic efficacy in ovarian cancer models. CRISPR-mediated KRAS knockdown in SKOV-3 cells dramatically altered three-dimensional spheroid morphology, reducing the average area six-fold, and significantly enhanced sensitivity to both cisplatin and paclitaxel in 3D cultures, where paclitaxel resistance was completely reversed. The Tet-ON system demonstrated dose-dependent chemosensitization with optimal effects at intermediate KRAS knockdown levels (~50–60%). PPRH oligonucleotides at sub-cytotoxic concentrations (50 nM) reduced cisplatin and paclitaxel IC_50_ values by approximately 50% in 2D cultures. Pharmacological KRAS inhibition with BI2865 produced striking synergy with paclitaxel (several hundred-fold sensitizations in 2D; complete reversal of 3D resistance), and additive effects with cisplatin. In KRAS-amplified Kuramochi cells (representing high-grade serous ovarian carcinoma), BI2865 enhanced paclitaxel efficacy, despite greater baseline chemoresistance. These findings establish KRAS as a promising chemosensitization target in ovarian cancer, with particular potential for taxane-based combination therapies.

## 1. Introduction

Ovarian cancer remains one of the most lethal gynecological malignancies, with an estimated 20,890 new cases and 12,730 deaths projected in the United States in 2025 [1]. The high mortality rate is attributable to late-stage diagnosis, with disease dissemination throughout the peritoneal cavity, and the frequent development of chemotherapy resistance [2,3]. Despite initial responsiveness to platinum-based chemotherapy and taxanes, roughly 85% of patients with advanced ovarian cancer experience disease recurrence, with median progression-free survival of approximately 18 months following primary treatment [4]. Although most patients initially exhibit platinum-sensitive disease, approximately 80% ultimately develop chemoresistance, at which point the disease becomes clinically intractable [3,5].

KRAS amplification is found in aggressive ovarian cancers, correlating with increased tumorigenicity, metastatic potential, and a poor prognosis [6,7]. KRAS is a 21 kD GTPase that plays a role in cell survival, proliferation, and differentiation [8,9]. It is constitutively expressed, but active only when GTP-bound. Normally functioning KRAS has a relatively short, and inducible, GTP-bound life. Mutations in RAS proteins are found in approximately one-third of all human tumors, with KRAS being the most frequently mutated isoform [10,11]. Mutation of the *KRAS* gene has been identified as a transforming oncogenic event, where it creates an unstable environment, allowing for more mutational selection and increasingly aggressive disease [12]. While activating KRAS mutations are less common in ovarian cancer compared to pancreatic or colorectal malignancies (occurring in approximately 6–10% of cases compared to >80% in pancreatic cancer and ~30–50% in colorectal cancer), aberrant KRAS signaling through upstream receptor tyrosine kinases and other oncogenic drivers is frequently observed in ovarian cancer [13,14]. Importantly, elevated KRAS expression and pathway activation, including KRAS amplification in high-grade serous ovarian cancer, have been implicated in chemotherapy resistance [15]. These resistance mechanisms operate through multiple pathways, including enhanced DNA repair capacity, altered apoptotic thresholds through modulation of anti-apoptotic proteins such as Bcl-2/Bcl-XL, and increased drug efflux via ATP-binding cassette transporters [16,17].

Recent advances in direct and indirect KRAS inhibition—including FDA-approved KRAS G12C inhibitors sotorasib (2021) [18] and adagrasib (2022) [19], investigational KRAS G12D inhibitor MRTX1133 [20], and pan-KRAS inhibitors BI-2865 (GDP-state) and RMC-6236 (GTP-state) [21,22]—have opened new therapeutic avenues to therapeutically target the KRAS pathway. The current study employs multiple complementary approaches—including gene editing, titratable molecular biology systems, targeted oligonucleotides modulating KRAS expression, and pan-KRAS inhibitors—to systematically investigate the potential of KRAS downregulation as a chemosensitization strategy in ovarian cancer using both two- and three-dimensional in vitro models. Our results demonstrate synergistic interactions between KRAS modulation and paclitaxel, representing a therapeutically exploitable vulnerability, with additive effects observed in combination with cisplatin. These findings support the clinical development of targeted KRAS downregulation or functional inhibition as a viable therapeutic approach for aggressive and metastatic ovarian cancers.

## 2. Results

### 2.1. Morphological and Chemo-Responsive Changes with KRAS Downregulation

#### 2.1.1. CRISPR-Based Gene Editing

**Figure 1:**
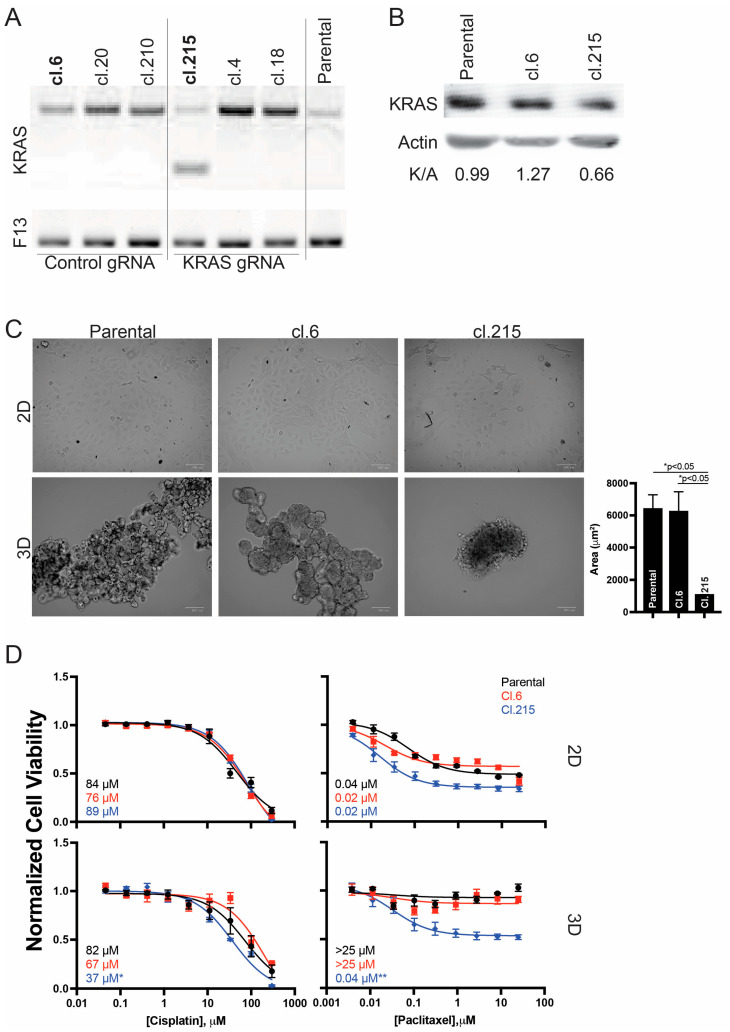
CRISPR/Cas9-mediated KRAS knockdown alters 3D morphology and enhances chemosensitivity in SKOV-3 ovarian cancer cells. (**A**) Confirmation of KRAS gene disruption in selected clones of SKOV-3 cells transfected with either control non-targeting gRNA (cl.6, cl.20, cl.210) or KRAS-targeting gRNA (cl.215, cl.4, cl.18); F13 serves as a loading control. Clone 6 (cl.6) was selected as the control with intact KRAS expression, and clone 215 (cl.215) was selected as the KRAS knockdown line with confirmed gene disruption. (**B**) Decreased expression was evaluated on the protein level by Western blot analysis of KRAS and actin (loading control) in parental SKOV-3 cells, cl.6 (control), and cl.215 (KRAS knockdown). Densitometric quantification expressed as KRAS/actin (K/A) ratios shows reduced KRAS expression in cl.215, compared to parental and cl.6. (**C**) Morphological effects of 2D and 3D conditions were evaluated. Representative phase-contrast microscopy images (scale bar = 100 µm) of parental, cl.6, and cl.215 cells cultured in 2D monolayer (top row) or 3D conditions (bottom row) after 8 days of growth. KRAS knockdown (cl.215) cells form significantly smaller, more compact 3D structures compared to parental and control cells, while 2D morphology remains unchanged (quantification on right). (**D**) Chemosensitivity to cisplatin (left column) or paclitaxel (right column) was examined in parental, cl.6, and cl.215 cells grown in 2D (top row) or 3D (bottom row) conditions. IC_50_ values are indicated for each condition. Error bars represent standard deviation from at least three independent experiments. KRAS knockdown (cl.215) in 3D conditions shows significantly (* *p* < 0.05 and ** *p* < 0.001, as compared to both parental and cl.6 cells) enhanced sensitivity to both cisplatin (IC_50_ = 37 µM vs. 82–89 µM) and paclitaxel (IC_50_ = 0.04 µM vs. >25 µM).

To examine the effects of KRAS modulation on ovarian cancer morphology and chemo-efficacy without confounding factors from targeted therapeutics, a CRISPR/Cas9 approach was utilized. Guide RNA was designed targeting the GC-rich KRAS promoter and SKOV-3 ovarian cancer cells were transfected with either KRAS-targeting gRNA or non-targeting control gRNA. Individual clones were isolated and screened for KRAS modulation. From the screened clones, clone 6 (cl.6) was selected as the control with intact KRAS expression, and clone 215 (cl.215) was selected as the modified target with confirmed disruption of the KRAS gene at the DNA level (Figure 1A) and decreased expression at the protein level (Figure 1B). Quantification of KRAS protein expression normalized to actin (K/A ratios) revealed that cl.215 exhibited substantially reduced KRAS levels (0.66) compared to both parental cells (0.99, representing a ~34% reduction) and cl.6 (1.27, representing a ~48% reduction).

We assessed the effect of KRAS downregulation on cell morphology in adherent (2D) and non-adherent (3D) culture conditions. No morphological effects were noted with any clone under 2D growth conditions; however, marked changes were observed with 3D growth parameters (Figure 1C). Qualitatively, parental and cl.6 cells formed loose, irregular aggregates with extended cellular projections and dispersed architecture. In contrast, cl.215 cells with knocked down KRAS grew in significantly smaller, more compact structures with smooth borders, suggesting altered cell–cell adhesion or matrix interaction properties. Quantitatively, cl.215 spheroids had an average area of 1054.5 ± 35.4 µm^2^ compared to 6386.9 ± 899.2 µm^2^ for parental cells (*p* = 0.019) and 6220.2 ± 1246.8 µm^2^ for cl.6 (*p* = 0.02), representing an approximately 6-fold reduction in spheroid size.

The panel of cells was further evaluated for sensitivity to the chemotherapeutic drugs cisplatin and paclitaxel in both 2D and 3D growth conditions (Figure 1D). Parental SKOV-3 cells displayed equivalent sensitivity to cisplatin across phenotypic growth conditions (IC_50_: 84 µM in 2D, 82 µM in 3D), consistent with the small molecule’s ability to penetrate denser 3D structures. Comparatively, paclitaxel only inhibited growth in the 2D phenotype (IC_50_: 0.04 µM), with no measurable activity up to 25 µM in 3D growth conditions, consistent with its larger size and hydrophobic nature limiting penetration into spheroid structures. No marked or significant changes in these response profiles were noted with control cl.6 (cisplatin IC_50_: 76 µM in 2D, 67 µM in 3D; paclitaxel IC_50_: 0.02 µM in 2D, >25 µM in 3D) or with cl.215 in 2D conditions (cisplatin IC_50_: 89 µM; paclitaxel IC_50_: 0.02 µM).

Notably, however, cl.215 in 3D growth conditions displayed markedly enhanced chemosensitivity. Cisplatin sensitivity significantly increased approximately 2.4-fold (IC_50_: 37 µM in 3D vs. 89 µM in 2D, *p* < 0.05), representing ~50% greater sensitivity compared to controls in 3D conditions. Most strikingly, an IC_50_ for paclitaxel could be determined in cl.215 3D cultures (0.04 µM), which was statistically significant (*p* < 0.001) compared to both parental and cl.6 cells in 3D conditions (both >25 µM), and was equivalent to the paclitaxel sensitivity observed in 2D growth conditions. These findings suggest that KRAS knockdown fundamentally alters 3D tumor architecture in ways that improve drug penetration and/or increase cellular vulnerability to chemotherapeutic agents, particularly for larger molecules like paclitaxel that typically show poor efficacy against spheroid cultures.

#### 2.1.2. Dose-Dependent Transcriptional Regulation of KRAS (Tet-ON System)

Following the observation that ~50% KRAS knockdown achieved via CRISPR technology produced notable effects on 3D morphology and chemoresponsiveness, a titratable Tet-ON inducible system was employed to examine the dose–response relationship between graded KRAS modulation, overall growth characteristics, and enhanced cisplatin responsiveness. SKOV-3 cells were sequentially transfected to generate stably integrated cell lines: first with a tetracycline-responsive transactivator (Tet-ON) plasmid, followed by double-stable transfection with a second plasmid, in which the expression of KRAS siRNA, or an empty cassette (control) was driven by a Tet-responsive promoter. This system enabled dose-dependent KRAS knockdown through titration of doxycycline concentration.

**Figure 2:**
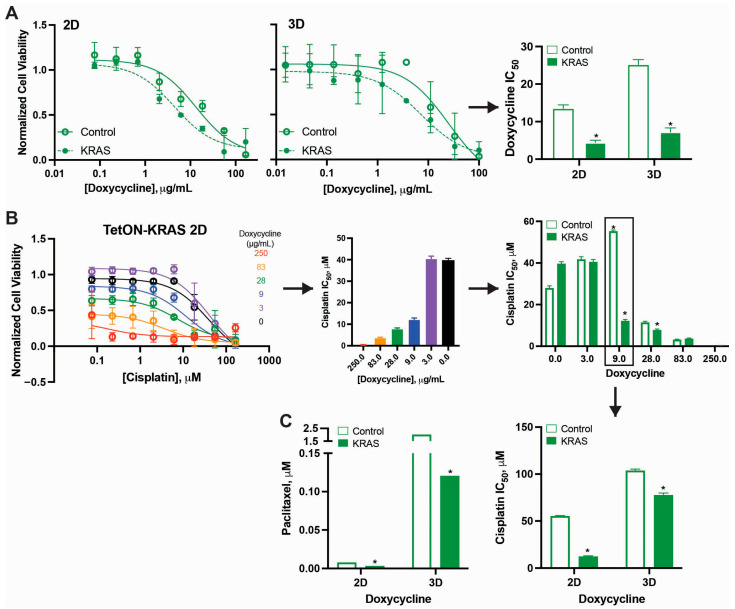
Tet-ON inducible KRAS knockdown system demonstrates dose-dependent effects on cell viability and cisplatin sensitivity. (**A**) SKOV-3 cells stably expressing Tet-responsive trans activator were transfected with either empty vector (control, open circles) or KRAS siRNA (KRAS, filled circles) under Tet-responsive promoter control. Left: 2D viability across doxycycline concentrations (0.01–100 µg/mL). Middle: 3D viability across doxycycline concentrations. Right: Bar graph showing doxycycline IC_50_ values in 2D and 3D conditions for control (clear bars) and KRAS knockdown (filled bars). KRAS knockdown cells show significantly enhanced sensitivity to doxycycline in both 2D (IC_50_ = 4.4 µM vs. 13.0 µM, * *p* < 0.05) and 3D (IC_50_ = 6.5 µM vs. 25.6 µM, * *p* < 0.05) conditions. (**B**) Cisplatin sensitivity was examined as a function of KRAS knockdown. Left: Representative cisplatin dose–response curves in Tet-ON KRAS 2D cultures at various doxycycline concentrations (0, 3, 9, 28, 83, 250 µg/mL, color coded as shown). Middle: Their correlating IC_50_’s. Right: Cisplatin IC_50_’s for control (clear bars) and KRAS knockdown (filled bars) across doxycycline concentrations in 2D conditions. Maximum cisplatin sensitization (4.6-fold relative to control, * *p* < 0.05) occurs at 9.0 µg/mL doxycycline, corresponding to approximately 50–60% KRAS reduction. (**C**) Significant changes in both cisplatin and paclitaxel IC_50_ are noted between control and KRAS knockdown induced by 9.0 µg/mL doxycycline in 2D and 3D grown cells. Error bars represent standard error of the mean from at least three independent experiments. * *p* < 0.05; statistical significance was determined by one- (**A**) or two-way (**B**,**C**) ANOVA with Tukey post hoc tests.

Cell viability in both control and KRAS knockdown lines was assessed across a range of doxycycline concentrations (0.01–100 µg/mL) in 2D and 3D culture conditions (Figure 2A). While control cells maintained relatively stable viability across most doxycycline concentrations, KRAS knockdown cells demonstrated significantly enhanced sensitivity to doxycycline-induced growth inhibition in both culture formats. In 2D conditions, the doxycycline IC_50_ decreased from 13.0 ± 1.5 µg/mL in control cells to 4.4 ± 0.8 µg/mL in KRAS knockdown cells, representing a 65% reduction (*p* < 0.05). This enhanced sensitivity was even more pronounced in 3D conditions, where the IC_50_ decreased from 25.6 ± 1.8 µg/mL in controls to 6.5 ± 1.5 µg/mL in KRAS knockdown cells, representing a 75% reduction (*p* < 0.05) (Figure 2A, right bar graph). These findings indicate that reduced KRAS expression renders cells more vulnerable to the growth-inhibitory effects of the induction system itself.

Cisplatin responsiveness was further examined across a gradient of doxycycline concentrations (0, 3.0, 9.0, 28.0, 83.0, and 250.0 µg/mL), which correspond to progressively increasing KRAS knockdown levels (Figure 2B). The Tet-ON-KRAS 2D panel shows representative dose–response curves for cisplatin at each doxycycline concentration, demonstrating a clear leftward shift (increased sensitivity) as doxycycline concentration increases, particularly in the KRAS knockdown line. Quantification of cisplatin IC_50_ values across the doxycycline titration revealed that cisplatin sensitivity increased in a dose-dependent manner for both control and KRAS siRNA-expressing cell lines (Figure 2B, right), likely reflecting some degree of baseline toxicity from the induction system. However, the magnitude of sensitization was substantially greater in KRAS knockdown cells.

Significant differences between control and KRAS-downregulating cell lines were most pronounced at lower to intermediate doxycycline concentrations. Most notably, at 9.0 µg/mL doxycycline, KRAS knockdown cells exhibited a 4.6-fold decrease in cisplatin IC_50_ compared to control cells at the same doxycycline concentration (Figure 2C, right, *p* < 0.05). At this doxycycline concentration, KRAS protein levels decreased by 62 ± 18% in 2D and 47 ± 25% in 3D phenotypes relative to uninduced conditions. The differential effect between control and KRAS knockdown persisted at 3.0 µg/mL doxycycline (*p* < 0.05) but diminished at higher doxycycline concentrations (28.0 µg/mL and above), likely due to the confounding cytotoxic effects of high-dose doxycycline in both cell lines.

Similar cisplatin sensitization effects were observed in 3D culture conditions at 9.0 µg/mL doxycycline, wherein the fold effect on cisplatin IC_50_ was 1.3-times greater in the KRAS-downregulating cell line compared to the control cell line. This observation is consistent with the general principle that 3D spheroid structures provide some degree of protection against chemotherapeutic agents. In combination treatments with paclitaxel in the 2D phenotype 2.5-fold sensitization was observed, and ~17-fold sensitization was noted with paclitaxel in the 3D phenotype, both in agreement with the CRISPR findings.

Together, these findings demonstrate that (1) the degree of KRAS knockdown can be titrated using the Tet-ON system, (2) even partial KRAS reduction enhances chemotherapy sensitivity in a dose-dependent manner, with optimal effects observed at intermediate knockdown levels (~50–60%), wherein a balance between KRAS knockdown and doxycycline inherent cytotoxicity is explored, and (3) this sensitization is particularly notable in 3D growth conditions and with paclitaxel, consistent with the earlier CRISPR-based observations.

#### 2.1.3. Promoter-Targeting PPRH Oligonucleotides

To further dissect the relationship between KRAS expression and chemotherapy sensitivity, we evaluate promoter-directed therapeutic oligonucleotides. Previous work from our laboratory identified lead polypurine reverse Hoogsteen hairpin (PPRH) oligonucleotide therapeutics that bind to the KRAS promoter and downregulate its expression [23]. Two leads, designated as PR and PPRH2, were shown to decrease SKOV-3 cell viability by approximately 50% at 100 nM concentrations. Building upon these findings, the present study evaluated whether non-cytotoxic doses of these KRAS-targeting PPRHs could sensitize ovarian cancer cells to standard chemotherapeutic agents.

**Figure 3:**
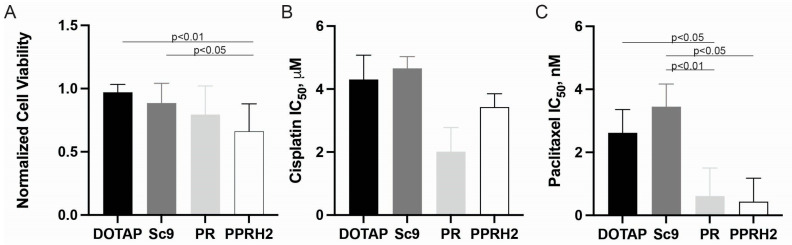
Polypurine reverse Hoogsteen hairpin (PPRH) oligonucleotides targeting KRAS enhance chemosensitivity in 2D cultures. (**A**) Transfecting SKOV-3 cells for 120 h with 50 nM oligonucleotides (DOTAP control, Sc9, PR, or PPRH2, as indicated by symbols) had moderate effects on cell survival, with PPRH2 demonstrating significantly decreased survival over both vehicle and scramble control. Co-treatment with 50 nM of each PPRH in the presence of increasing concentrations of cisplatin or paclitaxel in 2D culture conditions was also examined. (**B**) KRAS-targeting PPRHs (PR and PPRH2) reduce cisplatin IC_50_ from 4.6 µM to 2.1–3.4 µM (~50% reduction) and (**C**) paclitaxel IC_50_ from 2.6 nM to 0.6–0.7 nM (75–85% reduction), demonstrating chemosensitization. Error bars represent standard error of the mean. Note: 3D evaluation was not possible due to inefficient oligonucleotide delivery into dense spheroid structures.

Initial assessment of PPRH cytotoxicity at 50 nM concentrations confirmed minimal single-agent effects on cell viability. When delivered via lipid-based transfection (DOTAP), cells maintained normalized viability near 1.0, demonstrating that the transfection reagent alone had no significant cytotoxic effect. Similarly, treatment with 50 nM concentrations of the scrambled control oligonucleotide (Sc9), PR, or PPRH2 resulted in modest, non-significant reductions in viability. Specifically, Sc9 reduced viability to 0.89 ± 0.12, PR to 0.78 ± 0.13, and PPRH2 to 0.65 ± 0.21 relative to DOTAP control. While PPRH2 showed a trend toward greater single-agent activity, these differences were statistically significant only when comparing DOTAP to PPRH2 (*p* < 0.05) and Sc9 to PPRH2 (*p* < 0.01) (Figure 3A), indicating that this concentration was appropriately sub-cytotoxic for combination studies.

The chemosensitization potential of PPRHs was then evaluated by treating cells with 50 nM concentrations of Sc9, PR, or PPRH2 in combination with increasing doses of either cisplatin (1–100 µM) or paclitaxel (1–100 nM) in 2D culture conditions. For cisplatin, dose–response curves revealed that all oligonucleotide treatments, including the scrambled control, produced some degree of apparent sensitization compared to DOTAP alone (Figure 3B). However, the KRAS-targeting PPRHs (PR and PPRH2) demonstrated substantially greater effects. Quantitatively, cisplatin IC_50_ values decreased by approximately 50% in the presence of KRAS-targeting PPRHs: from 4.3 µM with DOTAP control and 4.6 µM with Sc9 scramble control to 2.1 µM with PR and 3.4 µM with PPRH2. The additive effects, particularly with PR, highlight the marked potential of specific and targeted KRAS downregulation in ovarian cancer.

Similar chemosensitization effects were observed with paclitaxel (Figure 3C). Treatment with PR or PPRH2 at 50 nM concentrations significantly enhanced paclitaxel efficacy, reducing the IC_50_ from 2.6 nM with DOTAP control and 3.5 nM with Sc9 scramble control to 0.6 nM with PR and 0.4 nM with PPRH2, representing approximately 75–85% reductions in IC_50_ values. Again, the scrambled control oligonucleotide Sc9 showed minimal effect on paclitaxel sensitivity, demonstrating the specificity of chemosensitization to KRAS-targeting sequences. Notably, the sensitization to paclitaxel observed with PPRHs contrasts with the lack of paclitaxel sensitization seen with CRISPR-mediated KRAS knockdown in 2D cultures (Figure 1D) but is in line with the Tet-ON system (Figure 2), suggesting that the kinetics and extent of KRAS modulation differ between these approaches.

As PPRH oligonucleotides require lipid-based transfection for cellular delivery, which is inefficient in dense 3D spheroid structures, evaluation of PPRH-mediated chemosensitization in 3D growth conditions was not technically feasible. Therefore, these experiments were restricted to 2D monolayer cultures.

Together, these findings demonstrate that KRAS-targeting PPRH oligonucleotides can enhance the efficacy of both platinum-based (cisplatin) and taxane-based (paclitaxel) chemotherapies at sub-cytotoxic concentrations, supporting the potential therapeutic utility of promoter-targeting oligonucleotides as chemosensitizing agents in ovarian cancer.

### 2.2. Enhanced Paclitaxel Efficacy with Pharmacologic KRAS Inhibition

#### 2.2.1. KRAS Inhibitors

To explore pharmacological approaches to KRAS inhibition, the pan-KRAS inhibitor BI2865 (targeting both mutant and wild-type KRAS) [24,25] and its proteolysis-targeting chimera (PROTAC) analog ACBI3 [22,26] were evaluated for their inherent cytotoxicity and chemosensitization potential in SKOV-3 cells cultured under both 2D and 3D growth conditions.

**Figure 4:**
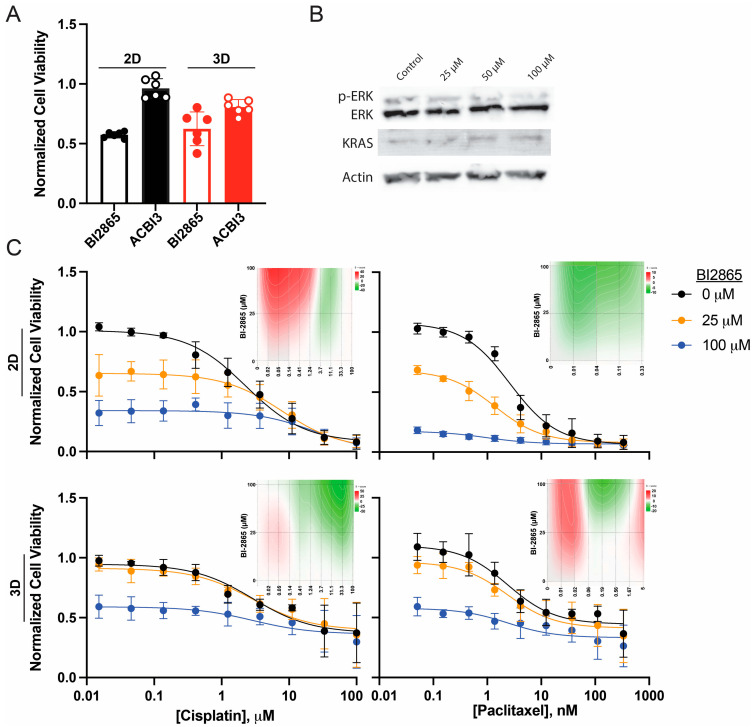
Pharmacological KRAS inhibition with BI2865 produces drug-specific chemosensitization in SKOV-3 cells. (**A**) Single-agent cytotoxicity of 100 μM KRAS inhibitor BI2865 (open bar) and PROTAC degrader ACBI3 (filled bar) for SKOV-3 cells in 2D (black bars) or 3D (red bars) cultures following 72 h treatment. BI2865 significantly reduces viability in both 2D and 3D conditions (*p* < 0.05), while ACBI3 shows minimal activity. Individual data points shown for n = 6 replicates; error bars represent standard deviation. (**B**) KRAS functional changes were observed following high doses of BI2865 in SKOV-3 cells for 72 h, with a decrease in ERK phosphorylation while KRAS expression increased. (**C**) SKOV-3 cells were co-treated with vehicle (0 µM, black circles), 25 µM (orange circles), or 100 µM (blue circles) BI2865 and cisplatin (left panels, 0.01–1000 µM) or paclitaxel (right panels, 0.001–1000 nM) for 72 h in 2D (top row) or 3D (bottom row) culture conditions. Dose–response curves show normalized cell viability; embedded graphs highlight dose-dependent ZIP scores with red indicating a tendency for synergism and green indicating a tendency for antagonism. Error bars represent standard error of the mean from at least three independent experiments.

Initial assessment of single-agent cytotoxicity revealed striking differences between the small molecule inhibitor and its PROTAC derivative (Figure 4A). At 100 µM concentration, BI2865 significantly reduced cell viability to approximately 0.55 ± 0.03 in 2D cultures and 0.60 ± 0.08 in 3D cultures, representing approximately 45% and 40% decreases in viability, respectively, compared to vehicle control (*p* < 0.05 for both conditions). In marked contrast, the PROTAC degrader ACBI3 showed minimal cytotoxic activity at the same concentration, maintaining viability near 1.0 (0.97 ± 0.05 in 2D and 0.87 ± 0.06 in 3D), which was not significantly different from untreated controls. This differential activity is likely attributable to the substantially larger molecular size of the PROTAC molecule (MW 1019 Da versus 466 Da for the small molecule inhibitor), which correlates with decreased cellular penetrance and potentially reduced target engagement. Given these limitations and the lack of significant single-agent activity, ACBI3 was not pursued for further combination studies.

BI2865 was evaluated for its ability to modulate KRAS function, but not KRAS expression, in a dose-dependent manner in SKOV-3 cells after 72 h incubation (Figure 4B). BI2865 is an inhibitor, but not degrader, of KRAS and thus was expected to not affect the expression of the oncogene. Notably, KRAS expression increased 2-fold with 100 μM BI2865 treatment, and ERK expression increased ~25%. Phospho-ERK cumulatively decreased only by 10%, but when accounting for the increased KRAS expression, overall function was decreased by 56%. The doses used to reach this level of inhibition and cytotoxicity are not clinically achievable, but studies continued in order to demonstrate proof of the principle.

BI2865 was subsequently evaluated for its ability to sensitize 2D- and 3D-cultured SKOV-3 cells to cisplatin and paclitaxel at sub-cytotoxic (25 µM) and cytotoxic (100 µM) concentrations (Figure 4C). In combination with cisplatin, BI2865 produced dose-dependent additive effects in both culture conditions. In 2D cultures (Figure 4C, upper left), co-treatment with 25 µM BI2865 resulted in a modest downward shift in the cisplatin dose–response curve, with cells maintaining approximately 60–65% viability at lower cisplatin concentrations compared to 100% without BI2865. The 100 µM BI2865 treatment produced more substantial effects, with baseline viability reduced to approximately 30–35% even at low cisplatin doses, reflecting the inherent cytotoxicity of BI2865 at this concentration. Zero Interaction Potential (ZIP) analyses were performed to underscore the combinatorial effects of these drugs. Low doses of cisplatin (<1 μM) were synergistic with all concentrations of BI2865, but higher concentrations tended towards antagonist in 2D conditions. Additive patterns (ZIP scores < 5) were observed in 3D cultures at low cisplatin concentrations (Figure 4B, lower left), while BI2865 at both concentrations shifted the cisplatin response curves downward but did not substantially alter the slope or IC_50_, consistent with independent mechanisms of action.

Paclitaxel efficacy was significantly, and apparently additively (ZIP scores <10), enhanced by BI2865 treatment in 2D cultures (Figure 4C, upper right). Without BI2865 treatment (0 µM), the paclitaxel IC_50_ was 2.8 nM. Co-treatment with 25 µM BI2865 decreased the IC_50_ to 0.5 nM, representing an approximately 5.6-fold sensitization. An amount of 100 µM BI2865 reduced the paclitaxel IC_50_ further but, as the combination was so toxic, determination of an IC_50_ was ineffective.

Importantly, and in concordance with the CRISPR-mediated KRAS knockdown findings (Figure 1D), BI2865 also significantly and synergistically enhanced paclitaxel cytotoxicity in 3D-cultured SKOV-3 cells (Figure 4C, lower right). While 3D spheroid cultures are typically resistant to paclitaxel (with the 0 µM BI2865 condition showing limited efficacy even at high paclitaxel concentrations), co-treatment with 100 µM BI2865 markedly sensitized cells to paclitaxel, decreasing the IC_50_ to 0.4 nM (*p* < 0.001). This represents one of the most striking findings of the study: pharmacological KRAS inhibition can overcome the intrinsic paclitaxel resistance conferred by 3D spheroid architecture. The 25 µM BI2865 concentration also showed enhanced paclitaxel activity in 3D cultures, though to a lesser extent than the 100 µM dose, suggesting a dose-dependent relationship between KRAS inhibition and paclitaxel sensitization.

The differential effects observed between cisplatin (predominantly additive) and paclitaxel (dominantly synergistic) combinations with BI2865 suggest distinct mechanistic interactions. The synergistic enhancement of paclitaxel efficacy, observed across multiple KRAS modulation approaches (CRISPR and Tet-mediated knockdown in Figure 1 and Figure 2, and pharmacological knockdown in Figure 3 or inhibition in Figure 4), points to a fundamental biological relationship between KRAS signaling and microtubule dynamics or cellular responses to microtubule-stabilizing agents. In contrast, the mostly additive effects with cisplatin suggest that KRAS inhibition and DNA-damaging chemotherapy operate through largely independent pathways, with combined cytotoxicity resulting from summation rather than true synergy.

These findings establish BI2865 as a promising chemosensitizing agent, particularly in combination with paclitaxel, and provide strong pharmacological validation of KRAS as a therapeutic target for overcoming chemoresistance in ovarian cancer, including in the clinically relevant 3D spheroid model.

#### 2.2.2. Validation in High-Grade Serous Kuramochi Ovarian Cancer Cells

While SKOV-3 cells represent the most studied ovarian cancer cell line and are derived from a serous adenocarcinoma, they have been shown to be less representative of high-grade serous ovarian carcinoma (HGSOC), which constitutes the most common (approximately 70% of cases) and most aggressive subtype of ovarian cancer [27]. To extend our findings to a more clinically relevant model, we evaluated Kuramochi cells, which have been identified as a cell line that more faithfully recapitulates HGSOC at both genetic and phenotypic levels [28,29]. Kuramochi cells harbor characteristic HGSOC mutations, including TP53 and BRCA2 inactivation, and notably exhibit copy number amplifications (CNAs) in both MYC and KRAS [28,29]. Specifically, Kuramochi cells possess more than 10-fold KRAS copy number amplification and demonstrate corresponding overexpression of KRAS at both the transcriptional and translational levels (proteinatlas.org [30]). This genetic context makes Kuramochi cells particularly relevant for evaluating KRAS-targeted therapeutic strategies.

**Figure 5:**
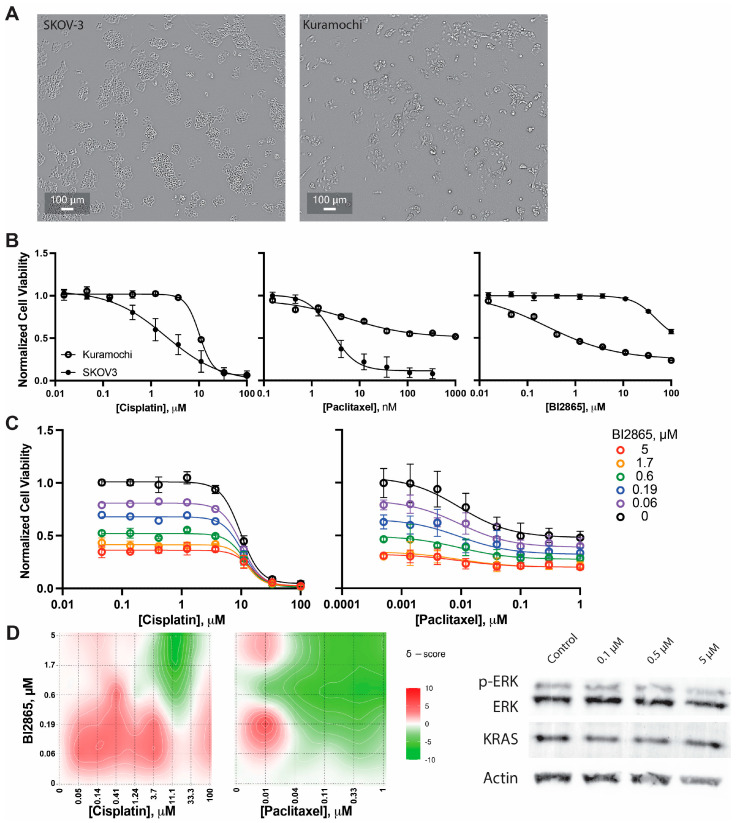
KRAS-amplified Kuramochi cells (HGSOC model) show differential chemosensitivity and reduced combination synergy compared to SKOV-3 cells. (**A**) Representative phase-contrast microscopy images (scale bar = 100 µm) of SKOV-3 (left) and Kuramochi (right) cells in 2D monolayer culture. Kuramochi cells exhibit larger cell bodies, more abundant cytoplasm, elongated spindle-like morphology, and decreased cell–cell clustering compared to the tight, epithelial-like clusters of SKOV-3 cells. (**B**) Dose–response curves comparing normalized cell viability of 2D Kuramochi (open circles) and SKOV-3 (filled circles) cells following treatment with cisplatin or paclitaxel. Kuramochi cells demonstrate ~5-fold greater cisplatin resistance and ~10-fold greater paclitaxel resistance compared to SKOV-3 cells. (**C**) Dose–response curves comparing normalized cell viability of Kuramochi (open circles) and SKOV-3 (filled circles) cells following treatment with BI2865 in 2D culture show ~4-fold enhanced sensitivity to BI2865, with response plateauing at ~50% viability, suggesting cytostatic rather than cytotoxic effects. Examination of protein expression changes in Kuramochi cells incubating with BI2865 for 96 h highlight an increase in KRAS expression and a decrease in KRAS function as evidenced by decreased ERK phorphorylation. Error bars represent standard error of the mean. (**D**) Combination effects of low-dose BI2865 with cisplatin or paclitaxel were evaluated in Kuramochi cells. (**E**) ZIP analyses show that BI2865 has predominantly additive effects with both agents but, unlike in SKOV-3 cells, synergistic enhancement is not observed. This suggests that KRAS amplification, TP53/BRCA2 mutations, and/or aggressive HGSOC phenotype may limit the magnitude of chemosensitization achievable with KRAS inhibition alone. Error bars represent standard error of the mean from at least three independent experiments.

Phenotypically, Kuramochi cells are morphologically distinct from SKOV-3 cells (Figure 5A). Kuramochi cells exhibit a more mesenchymal appearance with larger cell bodies, more abundant cytoplasmic content, and elongated spindle-like morphology. In monolayer culture, they display decreased cell–cell clustering and more dispersed growth patterns compared to the tight, epithelial-like clusters characteristic of SKOV-3 cells. These morphological features are consistent with a more aggressive, invasive phenotype.

Chemosensitivity profiling revealed that, while the literature reports have been inconsistent regarding Kuramochi drug responsiveness [28], under our experimental conditions, Kuramochi cells demonstrated resistance to standard chemotherapeutic agents compared to SKOV-3 cells (Figure 5B). The cisplatin IC_50_ in Kuramochi cells was approximately 10 µM, representing more than 5-fold higher resistance compared to SKOV-3 cells (IC_50_ ~2 µM). Similarly, Kuramochi cells showed an approximately 3.3-fold increase in paclitaxel IC_50_ (10 nM) compared to SKOV-3 cells (IC50 = 3 nM), with a decreased overall response. This enhanced chemoresistance is consistent with the aggressive clinical behavior of HGSOC and underscores the therapeutic challenge posed by this disease subtype.

In striking contrast to their chemoresistance, and in agreement with their KRAS amplification and overexpression profile, Kuramochi cells demonstrated enhanced susceptibility to KRAS inhibition with BI2865 (Figure 5C). The IC_50_ for BI2865 in Kuramochi cells was approximately 250 nM, compared to approximately 100 µM in SKOV-3 cells. Notably, the dose–response curve for BI2865 in Kuramochi cells appeared to plateau at approximately 25% viability rather than achieving complete cell death, suggesting a cytostatic response. This observation is consistent with KRAS dependency: cells with amplified KRAS may rely on this oncogene for proliferation and survival but may engage compensatory survival mechanisms.

The dose-dependent effect of BI2865 on the expression and function of KRAS was determined following 96 h of incubation (Figure 5C, right), consistent with both the cytotoxicity findings and the aforementioned study with SKOV-3. Interestingly, despite being an inhibitor, BI2865 mediated an increase in total KRAS protein expression of approximately 1.9-fold at the higher dose of 5 μM. In contrast, KRAS activity, as indicated by phosphorylated ERK (p-ERK) levels downstream of KRAS signaling, decreased by approximately 40% compared to untreated control cells. When normalized to the increase in total KRAS expression, this represents an approximately 50% reduction in KRAS-specific activity at 5 μM BI2865. To evaluate whether the chemosensitization effects observed in SKOV-3 cells extend to the HGSOC-representative Kuramochi model, cisplatin and paclitaxel were evaluated in combination with BI2685 at concentrations up to 5 µM (Figure 5D). This concentration range was selected to span the single-agent IC_50_ in Kuramochi cells to evaluate synergistic interactions (Figure 5E).

In combination with cisplatin (Figure 5D, left panel), BI2865 produced dose-dependent moderately additive (ZIP score <5) effects. In particular, cisplatin IC_50_ values non-significantly ranged from 9.8 µM with 0 µM BI2865 to 14.5 µM with 5 µM BI2865 and demonstrated additivity of two agents. The parallel downward shifts in the dose–response curves across all BI2865 concentrations (5, 1.7, 0.6, 0.19, and 0.06 µM) demonstrate that BI2865 reduces baseline viability in a dose-dependent manner without fundamentally altering cisplatin sensitivity. This finding is consistent with the additive cisplatin interactions observed in SKOV-3 cells and suggests that KRAS inhibition and DNA-damaging agents operate through independent mechanisms.

The additive effects of BI2865 were more pronounced in combination with paclitaxel (Figure 5C, right panel), with IC_50_ values dose-dependently decreasing from 10 nM without BI2865 to 3 nM with 5 µM BI2865, representing approximately 3.3-fold sensitization. However, careful analysis of the dose–response curves reveals that, unlike the synergistic shifts observed in SKOV-3 cells (Figure 4C), the enhancement in Kuramochi cells appears primarily additive rather than synergistic; the curves show roughly parallel shifts and ZIP scores range from −5 to +10 with low-dose combinations (Figure 5E).

The reduced magnitude of chemosensitization in Kuramochi cells compared to SKOV-3 cells may reflect several biological factors. First, the KRAS amplification in Kuramochi cells may create a scenario where even significant KRAS inhibition leaves residual KRAS activity sufficient to maintain downstream signaling. Second, the TP53 and BRCA2 mutations characteristic of Kuramochi cells may alter cellular responses to both chemotherapy and KRAS inhibition through mechanisms involving DNA damage response, cell cycle regulation, and apoptotic signaling. Third, the more mesenchymal and aggressive phenotype of Kuramochi cells may be associated with enhanced activation of compensatory survival pathways that limit the efficacy of combination therapy.

Despite the reduced synergistic potential compared to SKOV-3 cells, the demonstration that BI2865 can produce meaningful chemosensitization in the HGSOC-representative Kuramochi model—particularly the enhancement of paclitaxel efficacy—supports the translational potential of KRAS-targeted combination strategies. However, these findings also highlight that the magnitude and nature of chemosensitization may vary across ovarian cancer subtypes and genetic contexts, suggesting that patient selection based on KRAS expression levels, mutation status, and other molecular features may be critical for optimizing clinical responses to KRAS-targeted combination therapies.

## 3. Discussion

This study provides evidence that targeting KRAS represents a viable and potentially transformative strategy for enhancing chemotherapeutic efficacy in ovarian cancer, particularly in overcoming the intrinsic drug resistance associated with three-dimensional tumor architecture. Through complementary genetic (CRISPR/Cas9), inducible (Tet-ON), oligonucleotide-based (PPRH), and pharmacological (small molecule inhibitor) approaches to KRAS modulation, we have demonstrated that a reduction in KRAS expression or activity consistently sensitizes ovarian cancer cells to standard-of-care chemotherapeutic agents, though with important drug-specific and context-dependent differences.

Among the various KRAS-targeting strategies evaluated, the effects of stable CRISPR-mediated knockdown on 3D tumor architecture were particularly informative for understanding how KRAS shapes chemoresponsiveness. In particular, one of the most striking findings of the presented work is that CRISPR-mediated KRAS knockdown dramatically altered the three-dimensional growth characteristics of SKOV-3 cells, transforming them from large, loose, irregular aggregates with extended cellular projections into compact, dense spheroids approximately six-fold smaller in area. This morphological transformation has implications for drug delivery and efficacy wherein the dense, spheroid architecture typically confers significant resistance to chemotherapeutic agents due to limited drug penetration, hypoxic gradients, altered cell cycling, and enhanced cell–cell adhesion that promotes survival signaling. Paradoxically, KRAS knockdown cells in 3D culture demonstrated enhanced rather than diminished chemosensitivity, particularly to paclitaxel.

This unexpected finding suggests that KRAS modulation does more than simply reduce proliferation—it may reprogram the tumor microarchitecture to favor drug access and efficacy. The smaller, more compact spheroids formed by KRAS knockdown cells may have reduced diffusion distances for drug penetration, potentially explaining why paclitaxel, which typically shows poor efficacy in 3D cultures due to its large size and hydrophobic nature, became notably more effective following KRAS reduction. The fact that an IC_50_ for paclitaxel could be determined in KRAS knockdown 3D cultures (0.04 µM), comparable to 2D sensitivity, represents a near-complete reversal of 3D-mediated drug resistance and highlights the potential of KRAS-targeted strategies to address one of the fundamental challenges in solid tumor chemotherapy.

Beyond the noted morphological reprogramming, a broader pattern emerged when comparing the effects of KRAS modulation on the efficacy of different chemotherapeutic agents: combinations with paclitaxel demonstrated synergistic or highly potentiating effects, while combinations with cisplatin produced primarily additive effects. In SKOV-3 cells, proof-of-concept pharmacological KRAS inhibition with BI2865 achieved an enhancement of paclitaxel potency in 2D cultures and successfully overcame 3D resistance. Similarly, CRISPR-mediated knockdown and PPRH-based downregulation both significantly enhanced paclitaxel efficacy. In contrast, cisplatin sensitization was more modest across all approaches, with approximately 2-fold improvements in the best cases.

This differential pattern likely reflects fundamental differences in the mechanisms of action of these chemotherapeutic agents and their interactions with KRAS-dependent cellular processes. Paclitaxel stabilizes microtubules, preventing their dynamic instability and ultimately triggering mitotic catastrophe and apoptosis [31]. KRAS signaling influences multiple aspects of microtubule dynamics through downstream effectors including the RAF-MEK-ERK pathway [32,33], which regulates microtubule-associated proteins and spindle assembly checkpoint proteins, and the PI3K-AKT pathway, which affects microtubule stability through GSK3β and other targets [34]. KRAS also regulates the expression of β-tubulin isotypes and microtubule-associated proteins that determine cellular sensitivity to taxanes [35]. By reducing KRAS activity, cells may become more vulnerable to microtubule-stabilizing agents through multiple convergent mechanisms: altered tubulin isotype expression favoring taxane binding [36,37], reduced activation of survival pathways that normally counteract paclitaxel-induced apoptosis [38,39,40], and impaired spindle assembly checkpoint function [41,42] that would normally allow cells to escape mitotic arrest.

Cisplatin, in contrast, forms DNA adducts that trigger apoptosis through DNA damage response pathways [43,44] largely independent of KRAS signaling. While KRAS can influence cisplatin resistance through mechanisms including enhanced DNA repair capacity [45,46], increased glutathione synthesis by reducing oxidative stress [47,48], and activation of survival pathways that raise the apoptotic threshold [49,50], these effects appear to be less critical than the profound influence of KRAS on microtubule dynamics and mitotic processes. The additive rather than synergistic nature of KRAS inhibition plus cisplatin suggests that these agents kill cells through largely independent mechanisms, with combined cytotoxicity representing the sum of two separate insults rather than a multiplicative interaction.

The Tet-ON inducible system provided insight into the relationship between the degree of KRAS knockdown and chemosensitization. Maximal cisplatin sensitization (4.6-fold) was observed at intermediate doxycycline concentrations (9 µg/mL) that achieved approximately 50–60% KRAS reduction, rather than at the highest doxycycline concentrations that would presumably achieve maximal knockdown. This finding suggests that complete KRAS ablation may not be necessary—or even optimal—for chemosensitization, and that partial KRAS inhibition may achieve the best therapeutic window.

Several factors may contribute to this dose–response relationship. At very high doxycycline concentrations, the inherent cytotoxicity of the induction system itself seemed to create confounding effects that obscured or diminished the benefits of KRAS reduction. Although less likely, complete KRAS elimination may have triggered compensatory activation of alternative survival pathways or cellular stress responses that counteracted chemosensitization. Lastly, moderate KRAS reduction may be sufficient to disrupt critical downstream signaling nodes while avoiding the potential toxicities in non-cancerous tissues associated with complete KRAS loss. From a translational perspective, this finding is encouraging because it suggests that partial KRAS inhibition—which is more readily achievable with pharmacological agents and likely to have fewer on-target toxicities—may be sufficient for clinical benefit.

The extension of these studies to Kuramochi cells, which more faithfully represent high-grade serous ovarian carcinoma (HGSOC) due to their TP53 and BRCA2 mutations and KRAS amplification, revealed both validating similarities and important differences compared to SKOV-3 cells [28,29,30]. Kuramochi cells demonstrated the expected inverse relationship between KRAS dependency and chemosensitivity: they were approximately significantly more sensitive to KRAS inhibition, but several-fold more resistant to cisplatin and paclitaxel. This pattern strongly supports the concept that KRAS-amplified tumors are both more dependent on KRAS signaling (creating therapeutic vulnerability) and more resistant to conventional chemotherapy (creating therapeutic need).

The magnitude of chemosensitization achieved, however, by combining BI2865 with chemotherapy, was substantially reduced in Kuramochi cells compared to SKOV-3 cells. While paclitaxel sensitization in SKOV-3 cells was several hundred-fold with high-dose BI2865, Kuramochi cells showed only approximately 3.3-fold enhancement, and the interaction appeared primarily additive rather than synergistic. Several biological factors unique to Kuramochi cells and HGSOC may explain this differential response.

First, the 10-fold KRAS amplification in Kuramochi cells creates a much higher baseline level of KRAS protein [28,30], meaning that even substantial percentage inhibition may leave residual KRAS activity sufficient to maintain critical downstream signaling. The apparently cytostatic rather than cytotoxic response to BI2865 alone in Kuramochi cells (with viability plateauing around 25% rather than approaching zero) supports this interpretation and suggests that alternative or more potent KRAS inhibition strategies may be needed in KRAS-amplified contexts. Second, the TP53 and BRCA2 mutations that define HGSOC fundamentally alter cellular responses to both DNA damage and mitotic stress. TP53 loss eliminates a critical checkpoint that normally triggers apoptosis in response to cellular stress [51,52], potentially raising the threshold for chemotherapy-induced cell death and reducing the sensitization achieved by KRAS inhibition. BRCA2 deficiency, while creating vulnerability to PARP inhibitors and platinum agents through impaired homologous recombination repair, may also engage alternative DNA repair pathways and survival mechanisms [53,54] that limit the benefits of combination therapy. Third, the more mesenchymal, aggressive phenotype of Kuramochi cells may reflect the activation of epithelial–mesenchymal transition (EMT) programs associated with enhanced stemness, survival pathway activation, and drug resistance [55,56]. KRAS inhibition may be less effective at reversing these characteristics in cells where they are driven by multiple converging genetic alterations rather than primarily by KRAS signaling. Fourth, BI2865 is an “off-state” KRAS inhibitor that functions by shifting the conformational equilibrium toward the inactive, GDP-bound state [21]. However, in the context of KRAS amplification, the substantial overabundance of KRAS protein results in a larger fraction of molecules occupying the GTP-bound “on-state” at any given time, thereby maintaining enhanced downstream signaling despite inhibitor presence [57,58]. This mechanistic limitation suggests that “on-state” inhibitors, such as RMC-6236, which directly target the active GTP-bound conformation of KRAS [59], may prove more effective in KRAS-amplified contexts and represent a rational therapeutic strategy to be tested in future studies expanding these findings.

These findings have important implications for clinical translation: they suggest that while KRAS-targeted combination strategies show promise across ovarian cancer subtypes, the magnitude of benefit and the optimal combination partners may vary based on genetic context. KRAS-amplified tumors may require more aggressive KRAS inhibition strategies (such as KRAS degraders or combination with MEK inhibitors to achieve more complete pathway suppression), and tumors with BCRA mutations may benefit from the addition of agents that specifically target DNA repair mechanisms via PARP inhibition or restore apoptotic sensitivity.

This study employed four distinct approaches to KRAS modulation—CRISPR knockdown, Tet-ON inducible knockdown, PPRH oligonucleotides, and small-molecule inhibition—each with distinct characteristics relevant to therapeutic development. CRISPR-mediated knockdown achieved a stable 50% reduction in KRAS expression and produced the most dramatic effects on 3D morphology and paclitaxel sensitization but represents a genetic tool rather than a readily translatable therapeutic approach. The Tet-ON system allowed dose–response evaluation and confirmed that partial KRAS reduction is sufficient for chemosensitization, but the cytotoxicity of the doxycycline induction system at the concentrations needed for meaningful KRAS knockdown limits its utility beyond proof-of-concept studies.

PPRH oligonucleotides targeting the KRAS promoter [23] represent a potentially translatable approach that achieved significant chemosensitization with both cisplatin and paclitaxel in 2D cultures at sub-cytotoxic concentrations (50 nM). Notably, PPRHs produced paclitaxel sensitization in 2D conditions where CRISPR and Tet-ON approaches showed minimal effects, suggesting that promoter-targeted oligonucleotides may exert additional mechanisms beyond simple expression reduction—potentially including chromatin remodeling, altered transcriptional kinetics, or off-target effects on related genes. However, the requirement for lipid-based transfection significantly limits PPRH evaluation in 3D models and raises questions about in vivo delivery and tumor penetration that would need to be addressed before clinical development.

The small-molecule KRAS inhibitor BI2865 [21,24,25] emerged as the most promising approach from a translational perspective, achieving dose-dependent chemosensitization in both 2D and 3D cultures of both SKOV-3 and Kuramochi cells without requiring transfection or genetic modification. The dramatic synergy with paclitaxel in 3D SKOV-3 cultures—achieving an IC_50_ of 0.4 nM and effectively reversing 3D-mediated drug resistance—represents one of the study’s most clinically relevant findings. The lesser but still meaningful effects in Kuramochi cells (3.3-fold paclitaxel sensitization) suggest that BI2865 or similar agents could provide clinical benefit even in aggressive HGSOC tumors, though potentially requiring higher doses or combinations with additional pathway inhibitors.

The PROTAC degrader ACBI3 [26] showed minimal activity in this study, likely due to poor cellular penetration related to its large molecular size. However, the PROTAC approach remains promising for KRAS targeting, as newer-generation degraders with improved drug-like properties are in development. The ability of PROTACs to achieve more complete and sustained target elimination compared to competitive inhibitors could theoretically overcome some of the limitations observed with BI2865 in KRAS-amplified Kuramochi cells, though this remains to be tested.

The findings of this study implicate several interconnected mechanisms through which KRAS promotes chemoresistance and through which KRAS inhibition enhances chemosensitivity. At the morphological level, KRAS signaling promotes the formation of large, loose tumor aggregates with poor intercellular adhesion—architecture that limits drug penetration and creates microenvironmental niches protective against chemotherapy. KRAS reduction reverses this morphology, creating dense spheroids that, despite their compact nature, show enhanced drug sensitivity, possibly due to reduced diffusion distances and altered cell–cell signaling.

At the molecular level, KRAS-driven activation of the MAPK and PI3K pathways upregulates anti-apoptotic proteins (including BCL-2 family members, MCL-1, and survivin), enhances DNA repair capacity, modulates drug transporter expression, and alters cell cycle checkpoint function [50,60]—all of which raise the threshold for chemotherapy-induced cell death. KRAS also regulates expression of tubulin isotypes and microtubule-associated proteins that determine sensitivity to microtubule-targeting agents [35], providing a direct mechanistic link to the particularly striking synergy observed with paclitaxel. By inhibiting KRAS, cells are rendered more vulnerable to chemotherapy through multiple convergent mechanisms: reduced survival signaling, impaired DNA repair, altered drug transport, and increased susceptibility to mitotic catastrophe.

The apparent synergism with paclitaxel over cisplatin suggests that KRAS’s influence on microtubule dynamics and mitotic processes may be particularly critical for chemoresistance in ovarian cancer. This finding aligns with clinical observations that KRAS mutations or high KRAS expression in various cancers are often associated with taxane resistance [61], and suggests that KRAS-targeted therapies may be particularly valuable for overcoming taxane resistance and for re-sensitizing taxane-resistant tumors to this important drug class.

The consistent demonstration that KRAS modulation enhances chemotherapeutic efficacy across multiple experimental approaches and in both 2D and 3D culture systems provides strong preclinical rationale for the clinical evaluation of KRAS-targeted combination therapies in ovarian cancer. Ovarian cancers with KRAS amplification (as in Kuramochi cells) or activating KRAS mutations may be particularly suitable for KRAS-targeted combination approaches, as they demonstrate both greater KRAS dependency and greater baseline chemoresistance—creating both the opportunity and need for KRAS inhibition. Conversely, the finding that even wild-type KRAS SKOV-3 cells benefit from KRAS inhibition suggests that KRAS-targeted combinations may have broader applicability beyond KRAS-mutant or KRAS-amplified tumors, potentially extending to any ovarian cancer with high KRAS expression or pathway activation.

The dramatic and consistent synergy with paclitaxel across multiple experimental systems suggests that paclitaxel (or other taxanes such as docetaxel or nab-paclitaxel) should be prioritized as combination partners for KRAS inhibitors in ovarian cancer clinical trials. The current standard of care for ovarian cancer includes platinum/taxane doublet therapy [62,63,64,65], and the findings suggest that adding a KRAS inhibitor to this backbone could significantly enhance the taxane component while providing additive benefit with the platinum component. The Tet-ON findings suggesting that intermediate levels of KRAS inhibition (~50–60%) may be optimal for chemosensitization have important implications for dose selection and scheduling in clinical trials. Rather than pursuing maximum tolerated doses of KRAS inhibitors, it may be more effective to identify doses that achieve partial KRAS pathway suppression while minimizing toxicity. Furthermore, intermittent dosing schedules that allow for recovery of normal KRAS-dependent cellular functions between chemotherapy cycles might improve therapeutic windows.

Several limitations of this study warrant consideration. First, experiments were conducted in vitro, and future studies considering the tumor microenvironment, including stromal cells, immune cells, extracellular matrix, and vascular components [66,67], could significantly influence the efficacy of KRAS-targeted combinations in vivo. Patient-derived xenograft models and syngeneic mouse models [68,69] would provide important validation of these findings in more physiologically relevant contexts. Second, the study focused on two ovarian cancer cell lines, and extension to additional cell lines representing different histological subtypes, mutation profiles, and clinical behaviors would support the generalizability of the findings [29]. Third, the mechanisms underlying the drug-specific differences in chemosensitization were not directly investigated, and mechanistic studies examining effects of KRAS inhibition on microtubule dynamics, apoptotic signaling, DNA repair, and cell cycle regulation would provide valuable mechanistic insights.

Future studies will address these limitations while exploring several promising directions. The evaluation of KRAS inhibitor combinations with other relevant ovarian cancer therapies—including PARP inhibitors (particularly in BRCA-mutant contexts) [70], anti-angiogenic agents [71], and immunotherapies [72]—could identify additional beneficial combinations. Investigation of mechanisms of acquired resistance to KRAS inhibitor combinations, including potential bypass signaling pathways and adaptive transcriptional responses, would inform strategies to prevent or overcome resistance. The development of improved KRAS-targeting modalities, including KRAS degraders or downregulators with better drug-like properties, and combination approaches targeting multiple nodes in KRAS signaling pathways (such as SHP2-KRAS-MEK or KRAS-PI3K combinations), could enhance efficacy beyond what was achieved with single-agent BI2865.

Overall, this study provides comprehensive preclinical evidence that targeting KRAS represents a viable and potentially transformative strategy for enhancing chemotherapeutic efficacy in ovarian cancer. The consistent demonstration of chemosensitization across genetic, oligonucleotide-based, and pharmacological approaches to KRAS modulation, the dramatic reversal of 3D-mediated drug resistance, the particularly striking synergy with taxanes, and the proof-of-principle in HGSOC-representative Kuramochi cells collectively support the clinical development of KRAS-targeted combination therapies for ovarian cancer. While important questions remain regarding optimal patient selection, dosing strategies, and mechanisms of resistance, the magnitude and consistency of the observed effects suggest that KRAS inhibition deserves serious consideration as a chemosensitizing strategy in this challenging disease. As KRAS inhibitors continue to advance through clinical development for other indications, the findings presented here provide strong rationale for extending evaluation of these agents to ovarian cancer, with particular emphasis on combinations with taxane-based chemotherapy regimens.

## 4. Materials and Methods

### 4.1. Chemicals, Oligonucleotides and Plasmids

All chemicals listed were purchased from Sigma-Adrich (St. Louis, MO, USA), unless otherwise stated. Oligonucleotides (Table 1) were purchased from Eurofins Operon (Huntsville, AL, USA) as lyophilized DNA after salt-free dialysis. CRISPR/Cas9 plasmids were obtained from Addgene (Watertown, MA, USA), including the Cas9 sgRNA empty vector (Addgene plasmid #68463) from Su-Chun Zhang and pSpCas9(BB)-2A-Puro (PX459) V2.0 (Addgene plasmid #62988) from Feng Zhang. Tetracycline-inducible plasmids were purchased from Clontech through Takara (San Jose, CA, USA), specifically the Tet-On 3G inducible expression system (catalog #631168) and the pmRi-Zsgreen1 (catalog #631121) plasmids were utilized.

### 4.2. Plasmid Construction

Forward and reverse sequences (Table 1) were annealed (sgRNA F and sgRNA R, and siRNA F and siRNA R) and cloned into the appropriate plasmids using standard cloning techniques. Briefly, restriction enzymes (NEB Biolabs, Ipswich, MA, USA) were used, following the manufacturer’s instructions, to cut linear plasmids that were then separated on and extracted from 1% agarose gels; linear plasmids were incubated with annealed DNA, sealed with ligase, and re-circularized plasmids were separated on and extracted from 1% agarose gels.

### 4.3. Cell Lines and Cell Culture Conditions

SKOV-3 cells were purchased from ATCC (Manassas, VA, USA) and maintained in exponential growth in McCoys 5A medium (ATCC) supplemented with 10% fetal bovine serum and 1x penicillin/streptomycin at 37 °C in a humid incubator with 5% CO_2_. Kuramochi cells were purchased from FujiFilm Irvine Scientific (Santa Ana, CA, USA) and maintained in RPMI-1640 medium (ATCC) supplemented with 10% fetal bovine serum and 1x penicillin/streptomycin. Derivative SKOV-3 cell lines (clones 6 and 215, TET-On control and Tet-On KRAS) were maintained as described for the parental cells.

### 4.4. Derivative Cell Line Generation and Confirmation

SKOV-3 cells were dually transfected with 1 μg each of the Cas9 expression plasmid and either the empty or the KRAS sgRNA vectors using FuGene lipid reagent (Promega, Madison, WI, USA) at a 3:1 ratio. After 48 h, transfected cells were selected under puromycin (500 μg/mL) pressure and surviving cells were sub-cloned in 96-well plates at ~1 cell/plate. Surviving cells were expanded and genomic DNA was extracted (Roche Diagnostics, Indianapolis, IN, USA) from each clone and screened by PCR using the KRAS F and KRAS R primers, with amplification of the F13 gene (F13 R and F13 R primers) used as a loading control for effects on the KRAS promoter. Changes in protein expression were confirmed using Western blotting following standard procedures. Antibodies for KRAS (1:1000, catalog #3965) and actin (1:5000, catalog #4970) were from Cell Signaling (Danvers, MA, USA); antibodies used in subsequent studies to demonstrate KRAS function include ERK (1:1000, catalog #4695), phosphor-ERK (1:2000, catalog #4370) and fluorescently tagged secondary antibodies (AlexaFluor 488 anti-mouse and AlexaFluor 555 anti-rabbit, both from ThermoFisher, Waltham, MA, USA). Selected clones, as described in the manuscript, were expanded.

Similarly, SKOV-3 cells were transfected with the Tet-ON 3G plasmid using the FuGene system and cells were selected under G418 (2.5 μg/mL) pressure. Stable cells were secondarily transfected with GFP expressing empty control or KRAS siRNA-expressing pmRi-Zsgreen1 plasmids, and stable cell lines were selected with puromycin (500 μg/mL). Success of stable double transfection was confirmed microscopically with titration of tetracycline inducing dose-responsive GFP expression.

### 4.5. qPCR

Changes in mRNA were monitored by qPCR. In particular, SKOV-3 cells were seeded in tissue culture-treated 12-well plates at 10,000 cells per well or in 6-well ultra-low attachment plates at 10,000 or 200,000 cells per well, respectively. One or five days post-seeding, respectively, cells were treated with doxycycline at doses described in the text, for up to 72 h before cells were harvested and RNA was extracted using the GeneJet RNA purification kit (Roche Diagnostics, Basel, Switzerland) and quantified with a NanoDrop 2000 (Thermo Fisher Scientific, Waltham, MA, USA). An amount of 400 ng of RNA was reverse transcribed into cDNA using the qScript cDNA synthesis kit (Quantabio, Beverly, MA, USA) and used in multiplexed Taqman-based quantitative PCR with primers for KRAS (FAM-Hs00264282_m1) and GAPDH (VIC-Hs02786624_g1) from Thermo Fisher (Waltham, MA, USA) following the kit’s protocol. Expression was normalized to GAPDH and to untreated control using the 2^−ΔΔCt^ method; experiments were completed minimally in triplicate with technical duplicates on each qPCR plate run on a BioRad CFX96 (Hercules, CA, USA).

### 4.6. Cellular Viability Mono- and Combination Therapies

Cells were seeded in 96-well plates and allowed to either attach (2D) or form non-adherent clusters (3D) for up to 120 h before proceeding with treatments. SKOV-3 cells were seeded at 3000 cells per well for 2D tests and 10,000 cells per well for 3D studies; Kuramochi cells were seeded at 5000 cells per well for 2D tests. Drugs and compounds were incubated with the cells for 72 (SKOV-3) or 96 (Kuramochi) cells at the doses indicated in the above text; PPRH oligonucleotides were incubated with SKOV-3 cells for 120 h. At the termination of the studies, cells were incubated with 20 μL of activated CellTiter AQeuous (wth 5% phenazine methosulfate) for 2–4 h at 37 °C in a humid incubator with 5% CO_2_. Absorbance was measured at 490 nm on a VarioSkan plate reader, background was subtracted, and values were normalized to untreated controls. Non-linear regression of normalized values was performed using GraphPad Prism software (San Diego, CA, USA) to determine IC_50_ values. Experiments were performed minimally in triplicate with minimally technical duplicates.

### 4.7. Statistical Analyses

Statistical analyses were performed using GraphPad Prism 10 (GraphPad Software, San Diego, CA, USA). Data are shown as the mean ± SEM of all replicate experiments. Significance was determined as indicated in the figure legends; statistical changes in IC_50_ indicating additivity or synergy were determined with GraphPad Prism software using the extra-sum-of-squares F test with synergy indicated by significant changes (*p* < 0.05) in IC_50_ values using best-fit parameters of unshared parameters. Zero Interaction Potential (ZIP) analysis was performed using the synergy finder at www.synergyfinder.aittokallio.group (accessed 30 January 2026) [73]; ZIP scores indicate antagonism (negative numbers) or synergism (positive numbers) on a spectrum, with antagonism and synergy best indicated at scores > 10.

## Figures and Tables

**Table 1:** Oligonucleotides used in the current study.

Name	Sequence 5′-3′
PR	GGT GGA AGG GGC AGA AGA GAA AAG TTT TGA AAA GAG AAG ACG GGG AAG GTG G
PPRH2	GGG GAG AAG GAG GGG GCC GGG TTT TGG GCC GGG GGA GGA AGA GGG G
Sc9	AAG AAG AAG AAG AGA AGA ATT TTA AGA AGA GAA GAA GAA GAA
KRAS sgRNA F	CAC CGC CGC TCC TCC CCC GCC GGC C
KRAS sgRNA R	AAA CGG CCG GCG GGG GAG GAG CGG C
KRAS F	GAT GCG TTC CGC GCT CGA
KRAS R	AGT CCC TCC TCC CGC CAA
F13 F	GAG GTT GCA CTC CAG CCT TT
F13 R	ATG CCA TGC AGA TTA GAA A
KRAS siRNA F	TCG AGC TTG TGG TAG TTC CTG CTG GTG
KRAS siRNA R	GTA CCA CCA GCA GGA ACT ACC ACA AGC

## Data Availability

The original contributions presented in this study are included in the article. Further inquiries can be directed to the corresponding author.

## Abbreviations

The following abbreviations are used in this manuscript:

KRAS	Kirsten rat sarcoma viral oncogene homolog
3D	3-dimensional, non-adherent
2D	2-dimensional, adherent
gDNA	Guide RNA
siRNA	Small interfering RNA
CRISPR	Clustered regularly interspaced short palindromic repeats
Cas9	CRISPR-associated protein 9
PPRH	Polypurine Reverse Hoogsteen
Tet-ON	Tetracycline-inducible gene expression system
PROTAC	Proteolysis-targeting chimera
HGSOC	High-grade serous ovarian carcinoma
DOTAP	1,2-dioleoyl-3-trimethylammonium-propane
IC_50_	Half-maximal inhibitory concentration
PCR	Polymerase chain reaction
FDA	Food and Drug Administration
